# Forest carbon stocks and fluxes in physiographic zones of India

**DOI:** 10.1186/1750-0680-6-15

**Published:** 2011-12-25

**Authors:** Mehraj A Sheikh, Munesh Kumar, Rainer W Bussman, NP Todaria

**Affiliations:** 1 Department of Forestry and Natural Resources, HNB Garhwal University (A Central University), Srinagar Garhwal, Uttarakhand, India; 2 William L. Brown Centre, Missouri Botanical Garden, St. Louis, MO 63166-0299, USA

## Abstract

**Background:**

Reducing carbon Emissions from Deforestation and Degradation (REDD+) is of central importance to combat climate change. Foremost among the challenges is quantifying nation's carbon emissions from deforestation and degradation, which requires information on forest carbon storage. Here we estimated carbon storage in India's forest biomass for the years 2003, 2005 and 2007 and the net flux caused by deforestation and degradation, between two assessment periods i.e., Assessment Period first (ASP I), 2003-2005 and Assessment Period second (ASP II), 2005-2007.

**Results:**

The total estimated carbon stock in India's forest biomass varied from 3325 to 3161 Mt during the years 2003 to 2007 respectively. There was a net flux of 372 Mt of CO_2 _in ASP I and 288 Mt of CO_2 _in ASP II, with an annual emission of 186 and 114 Mt of CO_2 _respectively. The carbon stock in India's forest biomass decreased continuously from 2003 onwards, despite slight increase in forest cover. The rate of carbon loss from the forest biomass in ASP II has dropped by 38.27% compared to ASP I.

**Conclusion:**

With the Copenhagen Accord, India along with other BASIC countries China, Brazil and South Africa is voluntarily going to cut emissions. India will voluntary reduce the emission intensity of its GDP by 20-25% by 2020 in comparison to 2005 level, activities like REDD+ can provide a relatively cost-effective way of offsetting emissions, either by increasing the removals of greenhouse gases from the atmosphere by afforestation programmes, managing forests, or by reducing emissions through deforestation and degradation.

## Background

Concentration of atmospheric CO_2 _has accelerated upward during the past few decades. In the last decade, the average annual rate of CO_2 _increase was 1.91 parts per million (ppm). This rate of increase was more than double, as compared to the first decade of CO_2 _measurements at the Mauna Loa Observatory [1]. The implications of increased concentration of CO_2 _for climate and health of the global environment are topics of intense scientific, social and political concern. In contrast to economic globalization, no country can be left out of environmental globalization, as its consequences will sooner or later reach all. The direct solution to the problem is to reduce CO_2 _emission [2]. Forests absorb CO_2 _from atmosphere, and store carbon in wood, leaves, litter, roots and soil all acting as "carbon sinks". Carbon is released back into the atmosphere when forests are cleared or burned. Forests acting as sinks are considered to moderate the global climate. Overall, the world's forest ecosystems are estimated to store more carbon than the entire atmosphere [3].

Quantifying the substantial roles of forests as carbon stores, as sources of carbon emissions and as carbon sinks has become one of the keys to understanding and modifying the global carbon cycle. Thus, estimating carbon stock in biomass is the most critical step in quantifying carbon stocks and fluxes from the forests. Hence the focus of this paper is to estimate the carbon stocks in India's forest biomass, taking into account the inventory data for diversified forest types present in the country. Numerous ecological studies have been conducted to assess carbon stocks based on carbon density of vegetation and soils [4-6]. The results of these studies are not uniform and have wide variations and uncertainties probably due to aggregation of spatial and temporal heterogeneity and adaptation of different methodologies. IPCC [7] estimated an average carbon stock of 86 tonnes per hectare in the vegetation of the world's forests for the mid-1990s. The corresponding carbon in biomass and dead wood in forests was reported [8] to be 82 tonnes per hectare for the year 1990 and 81 tonnes per hectare for the year 2005.

India is a large developing country known for its diverse forest ecosystems and biodiversity. It ranks 10th amongst the most forested nations of the world [3] with 23.84 percent (78.37 million ha) of its geographical area under forest and tree cover [9]. With nearly 173,000 villages classified as forest fringe villages, there is obviously a large dependence of communities on forest resources. Thus, it is important to assess the likely impacts of emission from forests on climate change, to develop and implement adaptation strategies both for biodiversity conservation and protection and for safeguarding the livelihoods of forest dependent people [10].

Despite the importance of avoiding deforestation and associated emissions, developing countries have had few economic or policy incentives to reduce emissions from land use change [11,12]. 'Avoided deforestation' projects were excluded from the 2008-2012 first commitment period of the Kyoto Protocol because of concerns about diluting fossil fuel reductions, sovereignty and methods to measure emission reductions [13,14]. More recently the importance of including emissions reductions from tropical deforestation in future climate change policy has grown. The United Nations Framework Convention on Climate Change recently agreed to study and consider a new initiative, led by forest-rich developing countries, that calls for economic incentives to help facilitate reductions in emissions from deforestation and degradation in developing countries (REDD). The REDD has now become REDD plus and the core of REDD+ concept is to provide financial incentives to help developing countries voluntarily reduce national deforestation rates and associated carbon emissions below a baseline (based either on a historical reference case or future projection [10].

## Results

The estimated physiographic zone wise carbon stocks in biomass of Indian forests are as follows. The various factors used in this study are given in Table 1.

**Table 1:** Factors used in the present study

Parameter	Factor used
Mean wood density	0.7116
Mean biomass expansion factor	1.575
Ratio (below to above ground biomass)	0.266
Ratio (Other forest floor biomass)	0.15

Source: [47,35,32,10]

### Western Himalaya (WH)

This zone consists of Jammu and Kashmir, which covers the cold arid region of Leh (2000 m amsl) to the low altitude sub-tropical region of the southern plains (215-360 m amsl); Himachal Pradesh, which consists of high hills (temperate dry and wet parts) to mid hills and subtropical uplands, Uttarakhand hills, which consist of valleys, mid hills and high hills of the Western Himalayas and some parts of Punjab. The Western zone covers an area of 10.01% (329,255 km^2^) of the country with a forest area of 11.83% (91073 km^2^) for the year 2007 [9]. The total forest biomass stored in the western Himalayas was 1336.35, 1348.52 and 1397.58 Mt for the years 2003, 2005 and 2007 respectively (Table 2, 3 and 4). The increasing trend of carbon stock in biomass was also found for the years 2003, 2005 and 2007 with carbon stocks 601.35, 606.83 and 628.91 Mt respectively, with an incremental change of 5.48 Mt in ASP I and 22.08 Mt in ASP II (Table 5).

**Table 2:** Physiographic zone wise biomass details of India for the year 2003

**S.no**.	Physiographic zone	Growing stock (Mm^3^)	Biomass in Mt (AGB + BGB)	Forest floor biomass (Mt)	Total Forest biomass (80% of fresh weight) (Mt)
1	Western Himalayas	1159.880	1645.747	24.686	1336.346
2	Eastern Himalayas	549.354	779.474	11.629	632.932
3	North East	486.766	690.669	10.360	560.823
4	North Plains	284.986	404.365	6.065	328.344
5	Easter Plains	390.254	553.730	8.306	449.630
6	Western Plains	104.654	148.493	2.227	120.576
7	Central highlands	241.133	342.142	5.132	277.820
8	North Decan	372.991	529.234	7.938	429.738
9	East Decan	719.584	1021.014	15.315	829.063
10	South Decan	460.812	653.843	9.808	530.920
11	Western Ghats	556.057	788.986	11.835	640.656
12	Eastern Ghats	576.267	817.661	12.265	663.940
13	West Coast	260.443	369.540	5.543	300.066
14	East Coast	250.571	355.533	5.333	288.692

Total		6413.752	9100.438	136.506	7389.556

Source: The growing stock data had been taken from the FSI reports [9,28,29]

**Table 3:** Physiographic zone wise biomass details of India for the year 2005

**S.no**.	Physiographic zone	Growing stock (Mm^3^)	Biomass in Mt (AGB + BGB)	Forest floor biomass (Mt)	Total Forest biomass (80% of fresh weight) (Mt)
1	Western Himalayas	1170.444	1660.736	24.911	1348.518
2	Eastern Himalayas	567.834	805.696	12.085	654.225
3	North East	503.506	714.421	10.716	580.109
4	North Plains	283.251	401.903	6.028	326.344
5	Easter Plains	387.245	549.459	8.241	446.16
6	Western Plains	88.529	125.613	1.884	101.997
7	Central highlands	202.884	287.870	4.318	233.750
8	North Decan	339.121	481.176	7.218	390.715
9	East Decan	763.584	1083.445	16.252	879.758
10	South Decan	400.148	567.768	8.516	461.027
11	Western Ghats	572.588	812.442	12.187	659.703
12	Eastern Ghats	502.444	712.915	10.694	578.887
13	West Coast	243.158	345.014	5.175	280.151
14	East Coast	193.552	274.629	4.119	222.998

Total		6218.282	8823.088	132.346	7164.347

**Table 4:** Physiographic zone wise biomass details of India for the year 2007

**S.no**.	Physiographic zone	Growing stock (Mm^3^)	Biomass in Mt (AGB + BGB)	Forest floor biomass (Mt)	Total Forest biomass (80% of fresh weight) (Mt)
1	Western Himalayas	1213.03	1721.162	25.817	1397.583
2	Eastern Himalayas	542.55	769.820	11.547	625.094
3	North East	443.99	629.974	9.449	511.540
4	North Plains	246.87	350.282	5.254	284.429
5	Easter Plains	337.96	479.529	7.193	389.378
6	Western Plains	82.29	116.760	1.751	94.810
7	Central highlands	220.23	312.484	4.687	253.737
8	North Decan	364.54	517.243	7.759	420.002
9	East Decan	820.92	1164.799	17.472	945.817
10	South Decan	358.70	508.957	7.634	413.273
11	Western Ghats	580.46	823.610	12.354	668.771
12	Eastern Ghats	435.91	618.510	9.278	502.230
13	West Coast	254.08	360.513	5.408	292.737
14	East Coast	196.69	279.082	4.186	226.614

Total		6098.23	8652.746	129.791	7026.030

**Table 5:** Zone wise Carbon stock of India for 2003, 2005, 2007 and changes in ASP I and ASP II

S.no	Physiographic zone	Carbon stock in Biomass (Mt)			Change in carbon stock (ASP I)		Change in carbon stock (ASP II)	
		**2003**	**2005**	**2007**	**+ve**	**-ve**	**+ve**	**-ve**

1	Western Himalayas	601.35	606.83	628.91	5.48	-	22.08	-
2	Eastern Himalayas	284.82	294.40	281.30	9.58	-	-	13.1
3	North East	252.37	261.05	230.19	8.70	-	-	30.86
4	North Plains	147.75	146.85	127.99	-	0.9	-	18.86
5	Easter Plains	202.33	200.77	175.22	-	1.56	-	25.55
6	Western Plains	54.26	45.89	42.66	-	8.37	-	3.23
7	Central highlands	125.02	105.19	114.18	-	19.83	8.99	-
8	North Decan	193.38	175.82	189.00	-	17.56	13.18	-
9	East Decan	373.07	395.90	425.62	22.83		29.73	-
10	South Decan	238.91	207.46	185.97	-	31.45	-	21.49
11	Western Ghats	288.39	296.90	300.95	8.51		4.08	-
12	Eastern Ghats	298.77	260.50	226.00	-	38.27	-	34.5
13	West Coast	135.03	126.07	131.73	-	8.96	5.66	-
14	East Coast	129.91	100.35	101.98	-	29.56	1.63	-

	Total	3325.30	3223.96	3161.71	55.10	156.46	85.35	147.59

### Eastern Himalaya (EH)

This zone covers Sikkim, some part of West Bengal, and Arunachal Pradesh. It is characterized by hills, mountains and plateaus with near tropical to alpine climate conditions. The Eastern zone covers an area of 2.27% (74,618 km^2^) of the country with a forest area of 6.23% (47,965 km^2^) for the year 2007 [9]. The total forest biomass stored in the eastern Himalayas was 632.93, 654.22 and 625.09 Mt for the years 2003, 2005 and 2007 respectively (Table 2, 3 and 4). The carbon stock in biomass was found in increasing trend from 284.82 to 294.40 Mt during the year 2003 to 2005, with an incremental change of 9.58 Mt during Assessment Period first (ASP I) which thereafter has decreased to 281.30 Mt in ASP II, with a negative change of 31.1 Mt (Table 5).

### North east (NE)

This zone consists of 43-complete and 4-partial districts of north east part of the country in Arunachal Pradesh, Assam, Manipur, Meghalaya, Mizoram, Nagaland and Tripura. The North east zone (NE) cover nearly 4.08% (133,990 km^2^) of the total geographical area of country with a forest area of 10.32% (79,431 km^2^) and contribute 560.82, 580.11 and 511.540 Mt of biomass and 252.37, 261.05 and 230.19 Mt C reserves of the country for the years 2003, 2005 and 2007 respectively (Table 2, 3 and 4). There was an incremental (+ve) change of 8.70 Mt during the ASP I which have gone to a negative change of 30.86 Mt in ASP II (Table 5).

### Northern plains (NP)

The Northern Plains, also known as Indo- Gangetic plains, are the second largest zone after Western Himalayas and cover an area of 8.99% (295,780 km^2^) with a forest cover of 1.82% (13,992 km^2^). This zone consists of 102-complete and 8-partial districts from Chandigarh, Delhi, Haryana, Punjab, Uttar Pradesh and Uttarakhand. The northern plains are one of the most populous zones of the country resulting in continuous urbanization. The total forest biomass stored in the Northern Plains was 328.344, 326.344 and 284.43 Mt for the years 2003, 2005 and 2007 respectively (Table 2, 3 and 4). The decreasing trend of carbon stock in biomass was found for the years 2003, 2005 and 2007 with carbon stocks 147.75, 146.85 and 128.00 Mt respectively, with a negative change of 0.9 Mt during ASP I and 18.86 Mt in ASP II (Table 5).

### Eastern plains (EP)

This zone covers parts of Assam, Bihar and west Bengal. The Eastern zone covers an area of 6.79% (223,339 km^2^) of the country with forest area of 4.12% (31,709 km^2^) for the year 2007 [9]. The forest biomass stock in this zone was 449.630, 446.16 and 389.38 Mt for the years 2003, 2005 and 2007 respectively (Table 2, 3 and 4). The carbon stock stored in biomass was found in decreasing trend from 202.33 Mt in 2003, 200.77 Mt in 2005 and 175.22 Mt in 2007, with a negative change of 1.56 Mt during ASP I and 25.55 Mt in ASP II (Table 5).

### Western plains (WP)

A major part of Gujarat, part of Rajasthan and one district of Daman and Diu represents this region. According to FSI [9] this zone occupies 9.70% (319,098 km^2^) of the total geographical area of the country with a forest area of 1.78% (13,694 km^2^). The total forest biomass stored in this zone was 120.576, 101.997 and 94.810 Mt for the years 2003, 2005 and 2007 respectively (Table 2, 3 and 4). The carbon stock stored in biomass was found in decreasing trend from 54.26 Mt in 2003, 45.89 Mt in 2005 and 42.66 Mt in 2007, with a negative change of 8.37 Mt in ASP I which thereafter has decreased to 3.23 Mt in ASP II (Table 5).

### Central highlands (CHL)

This is the largest zone in terms of total geographical area and covers 11.36% (373,675 km^2^) of total geographical area of the country, with a forest area of 10.49% (80,788 km^2^). This zone comprises 52-complete and 19-partial districts, each one from Bihar, Gujarat and Haryana, Twelve from Uttar Pradesh 24 from Rajasthan and rest from Madhya Pradesh [9]. The total forest biomass stored in this zone were 277.820 Mt, 233.750 Mt and 253.737 Mt for the years 2003, 2005 and 2007 respectively (Table 2, 3 and 4), corresponding to carbon stock of 125.02 Mt, 105.19 Mt and 114.18 Mt with a negative change of 19.83 Mt during ASP I and a positive change of 8.99 in ASP II (Table 5).

### North Deccan (ND)

This zone covers a major part of Maharashtra and Madhya Pradesh and two districts namely Narmada and Vadodara from the Gujarat state. The North Deccan zone covers an area of 10.22% (355,988 km^2^) of the total geographical area of the country with forest area of 11.34% (87,260 km^2^) of the total forest area of the country [9]. The total biomass and carbon stock estimated for the years 2003, 2005 and 2007 was 429.738, 390.715, 420.002 Mt (Table 2, 3 and 4) and 193.38, 175.82 and 189.00 Mt (Table 5) respectively, with a negative change of 17.56 Mt in C stock during ASP I which showed a positive change of 13.18 Mt in ASP II.

### East Deccan (ED)

It covers major parts of Orissa, Jharkhand, Chhattisgarh, one complete and three partial districts of Madhya Pradesh, five partial districts from Bihar one district each from West Bengal (Purulia) and Uttar Pradesh (Sonbhadra) covering an area of 10.23% (336,289 km^2^) with a forest area of16.73% (128,757 km^2^) [9]. The carbon stock stored in biomass was found in increasing trend from 373.07, 395.90 and 425.62 Mt for the years 2003, 2005 and 2007 respectively, with an incremental change of 22.83 Mt and 29.73 Mt for ASP I and ASP II respectively (Table 5).

### South Deccan (SD)

This zone constitutes a major part of Karnataka state and some part of Andhra Pradesh with a total geographic area of 8.89% (292,416 km^2^) and having a forest area of 6.43% (49,451 km^2^) of the total forest area. The total forest biomass in this zone was 530.920, 461.027 and 413.273 Mt for the years 2003, 2005 and 2007 respectively (Table 2, 3 and 4), corresponding to carbon stock of 238.91, 207.46 and 185.97 Mt with a negative change of 31.45 and 21.49 Mt during ASP I and ASP II respectively (Table 5).

### Western Ghats (WG)

This zone covers an area of 2.20% (72,381 km^2^) of the total geographical area of the country with a forest area of 4.21% (32,399 km^2^) and represents the parts from Dadra & Nagar Haveli, Gujarat, Karnataka, Kerala, Maharashtra and Tamil Nadu [9]. The total biomass and carbon estimated for the years 2003, 2005 and 2007 was 640.656, 659.703, 668.771 Mt (Table 2, 3 and 4) and 288.39, 296.90, 300.95 Mt respectively, with an incremental change 8.51 Mt in ASP I and 4.08 Mt in ASP II (Table 5).

### Eastern Ghats (EG)

A major part of Andhra Pradesh and some parts of Orissa, Karnataka, and Tamil Nadu, covering an area of 5.83% (191,698 km^2^) with a forest cover 9.67% (74,418 km^2^). The total forest biomass in the Eastern Ghats was 663.940, 578.887 and 502.230 Mt for the years 2003, 2005 and 2007 respectively (Table 2, 3 and 4). The decreasing trend of carbon stock in biomass was also found with carbon stocks of 298.77, 260.50 and 226 Mt. There was a slight improvement in carbon loss which has decreased from 38.27 Mt in ASP I to 34.5 Mt in ASP II (Table 5).

### West coast (WC)

The west coast comprises the islands, Daman and Diu, Lakshadweep, Pondicherry and some parts from Goa, Gujarat, Karnataka, Kerala and Maharashtra, comprising 3.68% (121,242 km^2^) of the TGA with a forest area of 2.96% (20,736 km^2^). The total biomass stock in this zone was 300.066 Mt in 2003, 280.151 Mt in 2005 and 292.737 Mt in 2007 (Table 2, 3 and 4). The carbon stock decreased from135.03 Mt in 2003 to 126.07 Mt in 2005 with a negative change of 8.96 Mt, thereafter it has increased to 131.73 in 2007 with an incremental change of 5.66 Mt (Table 5).

### East coast (EC)

This zone is represented by Andaman and Nicobar Islands, parts of Pondicherry, Tamil Nadu, Orissa and Andhra Pradesh covering an area of 5.09% (167,494 km^2^) of the TGA of the country having a forest area of 2.32% (17,839 km^2^) and contribute 288.692, 222.998 and 226.614 Mt (Table 2, 3 and 4) of biomass and 129.9, 100.35 and 101.98 Mt C reserves of the country for the years 2003, 2005 and 2007 respectively. There was a negative change of 29.56 Mt during the ASP I which has increased to an incremental change of 1.63 Mt for ASP II (Table 5).

## Discussion

The significance of forest area as a single indicator of forest development has often been overemphasized-growing stock and carbon storage may be considered equally important parameters. The net exchange of carbon between terrestrial ecosystems and the atmosphere remains one of the most uncertain components of global carbon budget. The studies on net carbon release from Indian forests due to land use changes, shifting cultivation etc., have come up with divergent results. Of the fourteen physiographic zones of country, only Western Himalayas, East Deccan, and Western Ghats have consistently shown an incremental change in carbon stock in both the assessment periods. The continuous increase in the western Himalayas may be attributed due to dense vegetation, and less disturbance as these forests are located on mountains with low population density, whereas increase in carbon stock in East Deccan may be due to increase in forest cover which has increased from 128,006 km^2 ^in 2003 to 128,757 km^2 ^in 2007. The maximum CO_2 _sequestration was also found in this zone with an annual rate of 41.89 Mt CO_2 _yr^-1 ^in ASP I and 54.55 Mt CO_2 _yr^-1 ^in ASP II (Table 6).

**Table 6:** Physiographic zone wise "Gain" "loss" default values of CO_2 _and annual rate of CO_2 _changes

Physiographic zone	Atmospheric CO_2 _(Mt) (ASP I)		Atmospheric CO_2 _(Mt) (ASP II)		Annual rate of CO_2 _flux ASP I		Annual rate of CO_2_flux ASP II	
	
	Gain (+ve)	Loss (-ve)	Gain (+ve)	Loss (-ve)	Sequestration Mt yr^-1^	Emission Mt yr^-1^	Sequestration Mt yr^-1^	Emission Mt yr^-1^
Western Himalayas	20.11	-	81.03	-	10.05	-	40.51	
Eastern Himalayas	35.16	-	-	48.08	17.58	-	-	24.04
North East	31.92	-	-	113.25	15.96	-	-	56.62
North Plains	-	3.30	-	69.21	-	1.65	-	34.60
Easter Plains	-	5.72	-	93.80	-	2.86	-	46.9
Western Plains	-	30.72	-	11.85	-	15.36	-	5.92
Central highlands	-	72.78	32.99	-	-	36.39	16.50	-
North Decan	-	64.44	48.37	-	-	32.22	24.18	-
East Decan	83.78	-	109.10	-	41.89	-	54.55	-
South Decan	-	115.42	-	78.86	-	57.71	-	39.43
Western Ghats	31.23	-	14.97	-	15.61	-	7.48	-
Eastern Ghats	-	140.45	-	126.61	-	70.22	-	63.30
West Coast	-	32.88	20.77	-	-	16.44	10.38	-
East Coast	-	108.48	5.98	-	-	54.24	2.99	-

Total	202.22	574.19	313.23	541.66	101.11	287.09	156.61	270.83

Seven physiographic zones EH, NE, NP, EP, SD, WP, EG have shown negative change in carbon stocks, The maximum CO_2 _emissions was found in EG zone with an annual rate of 70.22 Mt CO_2 _yr^-1 ^in ASP I and 63.30 Mt CO_2 _yr^-1 ^in ASP II (Table 6). The continuous decrease in the carbon stock in these five zones is attributed due to rapid urbanization and industrialization, as these forests are in plains having high population density.

The decrease in carbon stock in North East zone is mainly due to shifting cultivation. Shifting cultivation practice has cleared 0.05 Mha of forest area every year in northeastern states of India and total 17.22 Mt wood biomass and 10.69 Mt C was removed at the rate of 1.72 Mt and 1.07 MtCyr^-1 ^respectively [15]. The old age practice of shifting cultivation has been a single responsible factor for the forest and land degradation, there by changing the land use pattern. Around 0.45 million families in north east region annually cultivate 10,000 km^2 ^forests where as the total forests area affect by jhumming is believed to be 44,000 km^2 ^[16].

The total carbon stock stored in forest biomass was 3325.30, 3223.96 and 3161.71 Mt with carbon density 42.93, 41.89 and 41.08 Ct ha^-1 ^for 2003, 2005 and 2009 respectively (Table 7). The total estimated carbon stock (wood only) reported by Manhas et al. [15] was 1085.06 Mt (or 1.09 Pg) and 1083.69 Mt (or 1.08 Pg) at a density of 24.94 and 24.54 t C ha^-1 ^for 1984 and 1994. Our estimated values are much higher than the values given earlier, which may be because these authors did not use the biomass expansion factor and did not consider the forest floor vegetation or it may be due increment in biomass due to increasing age structure of these forests. Kiswan et al. [10] have reported 2865.739 Mt of carbon stock in India's forest biomass for the year 2005 which is less than the present value (3223.96 Mt) calculated for 2005, which is due to the fact that these authors have considered 40% carbon in total biomass of country while we have considered 45% carbon in the total biomass. The carbon density t ha^-1 ^is within the ranged values given by Singh et al. [17] in their study of Central Himalayan forests. The carbon stock per unit area for Asian forests is 135, 90 and 40 t C ha^-1 ^(average, 88 t C ha^-1^) for moist, seasonal and open forests respectively [18], derived from wood volumes, and 250, 150 and 60 t C ha^-1 ^(average, 153 t C ha^-1^) for moist, seasonal and open forests respectively [4,18] based on direct measurements.

**Table 7:** Changes in forest cover, C stock, density in India

Year of assessment	Forest + Tree cover of India (%)	Total Carbon stock in biomass (Mt)	Carbon density of forests area (t ha^-1^)	Carbon density of TGA (t ha^-1^)
2003	23.57	3325.30	42.92	10.11
2005	23.41	3223.96	41.89	9.80
2007	23.84	3161.71	41.08	9.61

The carbon stock in India's forest biomass decreased continuously from 2003 onwards despite slight increase in forest cover of the country from 23.57% in 2003 to 23.84% in 2007 (Table 7). The reason for decreasing carbon stock may be due to substantial loss of high density growing stock forests, while new forest plantations or regenerated areas have low carbon densities, which results in increase in forest area and decrease in carbon stock. According to Richards and Flint [19], carbon storage in the vegetation in India from the year 1880 onwards shows a decreasing trend. The rate of carbon loss from the forest biomass in the ASP II had dropped by 38.27% in comparison to ASP I. This is mainly because the forest cover which earlier decreased from 23.57% in 2003 to 23.41% in 2005 thereafter increased to a record forest cover of 23.84% in 2007(Table 7). With the increase in forest cover the annual rate of CO_2 _emission had dropped from 185.99 Mt yr^-1 ^in ASP I to 114.21 Mt yr^-1 ^in ASP II (Table 8).

**Table 8:** Flux of CO_2 _(Mt) in India for ASP I and ASP II

Atmospheric CO_2 _(Mt)	"Gain"	"Loss"	Net Flux	Annual rate of Emission (Mt)
ASP I	202.22	574.19	371.98	185.99
ASP II	313.23	541.66	228.43	114.21

The total estimated CO_2 _loss from Indian forest biomass was 574.19 Mt at a rate of 185.99 Mt CO_2 _yr^-1 ^for ASP I and 541.66 Mt at rate 114.21 Mt CO_2 _yr^-1 ^for ASP II (Table 8). Each study in this connection has adopted a different approach based on different sources of data, different C pools for different years, resulting in net C flux that ranges from 0.4 Tg C yr^-1 ^[20] to a sink value of 5 Tg C yr^-1 ^[21]. The calculated carbon loss for Indian forests (wood only), reported by Manhas et al. [15] was 24.81 Mt C at a rate of 2.8 Mt C yr^-1 ^and 11.5 t C ha^-1 ^for the period of 1984 to 1994. Ravindranath et al. [21] reported that a total of 27.6 Mt C is emitted from the Indian forests annually as a result of deforestation and 12.87 Mt C from degraded forests. Houghton et al. [22] reported that forests hold more carbon per unit area in vegetation and soils than any other ecosystem that replaces them therefore conversion of forests into another land use also accompanies loss of biomass and carbon. Conversion of tropical forests to permanent agriculture and grazing lands has reduced the carbon density by 40%, whereas conversion to pasture has reduced the carbon content by 20% [23]. Houghton et al. [22] have reported that for 1980, approximately 80% of the net carbon flux from biota (2.0-2.5 Gt C yr^-1^) is associated with change in land use in the tropics. Defries et al. [24] on carbon emission from tropical deforestation and re-growth, based on satellite observation for the 1980s and 1990s, noted that for the 1990s total carbon flux from tropical deforestation and re-growth is 0.95 Gt C yr^-1^. The mean annual net C flux due to land use changes from Indian forests during 1880-1996 was estimated as 47 Tg C yr^-1^. The cumulative net carbon flux from Indian forests due to land use change (deforestation, afforestation, and phytomass degradation) was estimated as 5.45 Pg C. Dadhwal et al. [25] had estimated the long-term carbon emissions of 3.45 Pg C from fossil fuel use (coal, lignite, petroleum and natural gas) and industrial activity (cement manufacture) for India during the 20th century. The mean annual net C flux for the recent period (1985-1996) due to land use changes was estimated as 9.0 Tg C yr^-1^. This was an attempt to study the long-term pattern of net carbon flux from Indian forests with regional variabilities also, which has not been reported in earlier studies. The major decline in forest area could be accounted for a high net carbon release from Indian forests before the 1980 period. As a result of various afforestation programmes, forest conservation efforts, and joint forest management programmes by various government and non-government agencies, the forest area of India has stabilized to approximately 64 Mha, during the recent period, i.e., after 1980 [26]. The increase in forest area resulted in little uptake of carbon in Indian forests. For the recent period the Indian forests are a small source of carbon, which compares favorably with peak net annual carbon emissions during 1970-1980s [26]. The rate of afforestation in India is 2 Mha per annum, which is considered to be one of the highest among the tropical countries [27].

The Bali Action Plan included all the essentials of forest improvement i.e. reducing deforestation, conservation and sustainable management and enhancement of forest carbon stocks. Whether forests act as reservoirs, sinks for carbon from the atmosphere, or sources of GHGs depends on several factors such as the age of the forest, the management regime, other biotic and abiotic disturbances (e.g. insect pests, forest fires, etc.) and human-induced deforestation. Planting forests (afforestation and reforestation) clearly provides an opportunity to sequester carbon in vegetation and soils. However, it takes decades to restore carbon stocks that have been lost as a result of land-use changes. The reduction of deforestation and enhancement of forest carbon stocks are the two sides of the same coin, where one cannot do without the other. Both are equally important.

## Conclusion

In the present study an attempt was made to estimate the carbon stock of the country for two assessment periods, because estimating forest biomass carbon is the most critical step in quantifying carbon stocks and fluxes from forests. The carbon stock in India's forest biomass decreased continuously from 2003 onwards, despite slight increase in forest cover. Increasing forest cover will not help in REDD implementation unless deforestation and degradation will not be reduced because country's forest cover has already been degraded and dense forests are losing their crown density and productivity continuously. REDD implementation will be a challenge for India because of the complexity of the different elements influencing deforestation and forest degradation and requires a range of policy approaches and positive incentives to address the challenges. However with the rate of carbon loss from the forest biomass in the ASP II had dropped by 38.27% in comparison to ASP I. This is mainly because the forest cover which earlier decreased from 23.57% in 2003 to 23.41% in 2005 thereafter increased to a record forest cover of 23.84% in 2007 It was observed that with the increase in forest cover of the country the CO_2 _emission from the forestry sector had also started decreasing. With the Copenhagen Accord, India along with other BASIC countries China, Brazil and South Africa is voluntarily going to cut emissions. India will voluntary reduce the emission intensity of its GDP by 20-25% by 2020 in comparison to 2005 level. Activities like REDD+ can provide a relatively cost-effective way of offsetting emissions, either by increasing the removals of greenhouse gases from the atmosphere by afforestation programmes, managing forests, or by reducing emissions through deforestation and degradation.

## Methods

The total standing above-ground biomass of woody vegetation is often one of the largest carbon pools. The above-ground biomass comprises all woody stems, branches, leaves of living trees, creepers, climbers, and epiphytes as well as herbaceous undergrowth. Estimation of carbon stocks stored in Indian forests, in the present study is based on the secondary data of growing stock data published by Forest survey of India [28,29,9] in the State of Forest Reports. Assessment of biomass was based on the consideration that all lands, more than one hectare in area, with a tree canopy density of more than 10 per cent are defined as 'Forest'. The satellite data used for 2003 related to the period from 2001 to 2003, the data for the 2005 assessment related to the period from 2003 to 2005, and for 2009, pertained to 2006 to 2007.

Suitable biomass increment values (expansion and conversion for calculating total tree above ground biomass) and the ratio of below and above ground biomass (for calculating total tree biomass above and below ground) as available in different studies covering a range of forest types of the country were used in the present study. The various factors used in this study are given in Table 1.

Estimation of carbon stocks present in biomass is based either on IPCC (Good Practice guidelines (IPCC, GPG, 2003) or published literature for conversion and other factors starting from the growing stock (GS) data of forest inventories. The biomass in this study was calculated as;

$$\text{AGB}={\text{G}}_{\text{stk}}\times \text{MD}\times {\text{B}}_{\text{exf}}$$

Where,

AGB Above Ground Biomass Mt

G_stk _= Growing Stock in Mm^3^

MD = Mean density

B_exf _= Biomass expansion factor

The Below Ground Biomass was calculated by root shoot ratio:

$$\text{BGB}=\text{AGB}\times {\text{R}}_{\text{bel}.\text{ab}}$$

Where,

BGB = Below Ground Biomass Mt

R_bel.ab _= Ratio Below to Above Ground Biomass

The total biomass was estimated as;

$${\text{T}}_{\text{bm}}=\text{AGB}+\text{BGB}$$

In general, other forest floor biomass accounts for less than 2 percent of total biomass of closed forest formations [30,31]. However for this study, ratio was adopted based on the published records for different vegetation types and different localities, and also keeping in view its application and representation for the country level estimates [32-35]. The forest floor biomass was estimated by the following.

$${\text{F}}_{\text{fb}}={\text{T}}_{\text{bm}}\times {\text{R}}_{\text{tbm}}$$

Where,

F_fb _= forest floor biomass in Mt

T_bm _= Total biomass in Mt

R_tbm _= Ratio to total biomass in Mt

Total forest biomass was estimate as;

$$\text{TFB}={\text{T}}_{\text{bm}}+{\text{F}}_{\text{fb}}$$

To estimate the total dry weight in biomass 80% of total forest biomass was considered. Biomass material contains about 40% carbon by weight. The variability of approximately 9% depends on the nature of the biomass material [36,37] although most studies have used the carbon proportions between 40 to 50% depending on the requirements [38-43].

The carbon content of vegetation is surprisingly constant across a wide variety of species. Most of the information for carbon estimation described in the literature suggests that carbon constitutes between 45 to 50 percent of dry matter [44,45]. To estimate the total amount of carbon stocked in India's forests, dry weight of biomass was converted into carbon by multiplying with a factor of 0.45 as used by Woomer [46].

The change in Carbon stocks was assessed by the stock change method as per IPCC guidelines.

$$\Delta C=C_{2}-C_{1}$$

ΔC = Change of carbon stock

C_2 _= Carbon stock at time 2

C_1 _= Carbon stock at time 1

For annual change in C stocks following equation were used.

$$\Delta C=\left(C_{2}-C_{1}\right)∕(t_{2}-t_{1})$$

One ton of carbon in wood or forest biomass represents 3.67 tons of atmospheric carbon dioxide. Total atmospheric CO_2 _accumulated or emitted by the forest biomass was estimated by multiplying the carbon Stock values with 3.67, the molecular weight of Carbon dioxide. The total carbon stock change in India was estimated by Gain loss (default) method.

$$\Delta C=\Delta C_{\text{G}}-\Delta C_{\text{L}}$$

## Competing interests

The authors declare that they have no competing interests.

## Authors' contributions

MAS, MK and NPT share the contributions to data analysis, and compilation of this manuscript. RWB shares his valuable contribution from initial manuscript drafting to final submission. All authors read and approved the final manuscript.
